# Water consumption and prevalence of irritable bowel syndrome among adults

**DOI:** 10.1371/journal.pone.0228205

**Published:** 2020-01-24

**Authors:** Asma Salari-Moghaddam, Ammar Hassanzadeh Keshteli, Ahmad Esmaillzadeh, Peyman Adibi

**Affiliations:** 1 Students' Scientific Research Center, Tehran University of Medical Sciences, Tehran, Iran; 2 Department of Community Nutrition, School of Nutritional Sciences and Dietetics, Tehran University of Medical Sciences, Tehran, Iran; 3 Department of Medicine, University of Alberta, Edmonton, Alberta, Canada; 4 Integrative Functional Gastroenterology Research Center, Isfahan University of Medical Sciences, Isfahan, Iran; 5 Obesity and Eating Habits Research Center, Endocrinology and Metabolism Molecular-Cellular Sciences Institute, Tehran University of Medical Sciences, Tehran, Iran; 6 Department of Community Nutrition, School of Nutrition and Food Science, Isfahan University of Medical Sciences, Isfahan, Iran; University of Malaya Faculty of Medicine, MALAYSIA

## Abstract

**Background and aim:**

No previous study examined the association between whole-day water intake and odds of irritable bowel syndrome (IBS). We examined the association between whole-day water intake and odds of IBS in a large sample of Iranian adults.

**Methods:**

This cross-sectional study was conducted among 4763 adults in Isfahan, Iran. Daily water intake was assessed using a pre-tested questionnaire by asking questions about the average number of glasses of water consumed in a day. IBS was assessed and defined using a modified Persian version of Rome III questionnaire.

**Results:**

After taking potential confounders into account, no significant association between water consumption and odds of IBS was seen (OR: 1.14; 95% CI: 0.74–1.78). We observed that participants who were taking >8 glasses/d of water had greater odds of IBS-M in comparison to those who consumed <2 glasses daily (OR: 2.07; 95% CI: 1.01–4.22). No significant association was observed between water intake and odds of IBS in either gender as well [for men: OR: 1.15; 95% CI: 0.59–2.24 and for women: OR: 1.15; 95% CI: 0.62–2.12]. By BMI status, no significant association was observed between whole day water intake and odds of IBS among normal weight (BMI<25 kg/m^2^) or overweight and obese people (BMI≥25 kg/m^2^).

**Conclusion:**

We found that whole-day water intake was not associated with odds of IBS. A significant association between consumption of >8 glasses of water per day and odds of IBS-M was observed.

## Introduction

Irritable bowel syndrome (IBS) is a chronic functional gastrointestinal disorder characterized by abdominal pain and altered bowel habits [1]. It is thought to be a multifactorial disorder, in which dysregulation of the hypothalamic-pituitary-adrenal axis, neuroendocrine alterations and visceral hypersensitivity are involved. The hallmark symptoms in this condition are abdominal pain and disordered gut motility [2]. Although IBS is not a life-threating condition, patients with IBS have reduced quality of life [3]. In addition, IBS imposes a high economic burden to the family and public health system, such that in USA, the direct cost for diagnosis and control of this condition is between $1.7 and $10 billion annually [4].

Lifestyle-related factors have long been investigated in relation to IBS. For instance, previous studies have shown that women with irregular meal pattern had greater odds of IBS. In addition, heavy intra-meal fluid consumption was protectively associated with odds of IBS [5]. Adherence to a regular meal pattern and confine the intake of alcohol, caffeine, spicy foods and fat can be an effective strategy for IBS management [6, 7]. In addition, sufficient hydration is recommended for IBS patients [8]. Although there is no information on the association between water intake and IBS, the association of water consumption with other chronic diseases has been examined in previous studies. For instance, high total water intake was protectively associated with chronic kidney disease [9]. High intake of water was also prospectively associated with increased risk of mortality among women, but not men [10].

To our knowledge, there is no previous study that examined the association between whole-day water intake and odds of IBS. Water intake might be associated with improvement of IBS through affecting GI function. Water intake might improve constipation among IBS-C patients. In addition, drinking water is a common suggestion for IBS-D patients to prevent diarrhea-induced dehydration. However, it is not clear whether water intake can relieve abdominal pain among IBS patients. The aim of this study was, therefore, to examine the association between whole-day water intake IBS and its severity in a large sample of Iranian adults.

## Methods and materials

### Study population

This cross-sectional study was conducted within the framework of the Study on the Epidemiology of Psychological, Alimentary Health and Nutrition (SEPAHAN) project, a cross-sectional study that investigated the prevalence of functional gastrointestinal disorders (FGIDs) and their relationship with lifestyle factors. Details of SEPAHAN project have been published elsewhere [11]. Inclusion criteria for this study were as follow: Iranian general adults working in 50 different healthcare centers affiliated to Isfahan University of Medical Sciences (IUMS) across Isfahan province. In this project, data were collected in two main phases between April 2010 and May 2010. To calculate required sample size, we hypothesized the prevalence of FGDIs as 15% in Iran [11]. Considering the study power of 80% and type 1 error of 5% and desired confidence interval of 0.03, the minimum required sample size for the current study was estimated to be 1387 subjects based on suggested formula for cross-sectional studies. To collect information about anthropometric indices, demographic and lifestyle factors, including dietary intakes and physical activity, self-administered questionnaires were distributed among 10,087 subjects aged 18–55 years in the first phase, and 8691 participants returned the completed questionnaires (response rate: 86.16%). In the second phase, 9652 questionnaires were distributed and 6239 completed questionnaires were returned (response rate: 64.64%). We used national identification numbers to match the questionnaires from both phases. We were unable to match 1476 questionnaires in the second phase with those in the first phase due to the lack of national identification number. Finally, we were able to match 4763 questionnaires in the second phase with their corresponding questionnaires in the first phase. All participants provided informed written consent form. The study protocol was ethically approved by the Regional Bioethics Committee of Isfahan University of Medical Sciences (#189069, #189082, and #189086).

### Assessment of water intake

To examine total daily water intake in routine life, participants were requested to report the average number of glasses of water they usually consume in a day. The possible choices to this response were <2, 2–5, 6–8 and >8 glasses of water during the whole day.

### Assessment of IBS

A modified Persian version of the Rome III questionnaire, as part of the main comprehensive questionnaire, was used for assessment of IBS. During the face validation of the questionnaire, we found that most participants were unable to distinguish between the descriptors used in the original Rome III questionnaire (never, less than 1 day a month, 1 day a month, 2–3 days a month, 1 day a week, more than 1 day a week, every day). We, therefore, modified the rating scales to consist of only four descriptors (i.e., never or rarely, sometimes, often, and always). We also decided to ask about the presence of each symptom in the past 3 months instead of questioning patients about the beginning of each symptom in more than 6 months prior to the evaluation, which already exists in original ROME III questionnaire. IBS was defined according to ROME III criteria as having recurrent abdominal pain or discomfort, at least sometimes, in the last 3 months associated with two or more of these criteria: improvement with defecation at least sometimes and onset associated with change in frequency or form (appearance) of stool, at least sometimes. IBS with constipation (IBS-C) was defined as having IBS and both of the following criteria: (i) hard or lumpy stools at least sometimes and (ii) lack of loose, mushy or watery stools. IBS with diarrhea (IBS-D) was defined as having IBS and both of the following criteria: (i) lack of hard or lumpy stools and (ii) loose, mushy or watery stools at least sometimes. Mixed IBS (IBS-M) was defined as having IBS and both of the following criteria: (i) hard or lumpy stools at least sometimes and (ii) loose, mushy or watery stools at least sometimes.

### Assessment of other variables

Required information on other variables including age, sex, marital status, smoking status, and use of supplements and medications was obtained using a self-administered questionnaire. Meal pattern regularity was also assessed through asking individuals the following question: “How often do you consume your meals regularly?” Subjects were able to choose one of these choices: never, sometimes, often or always. Individuals who had reported that never or sometimes consume meals regularly were defined as having irregular meal pattern. Dental status was also examined and subjects were categorized as “having all teeth”, “lost 1–5 teeth” and “lost >5 teeth”. Depression was assessed using the Iranian validated version of Hospital Anxiety and Depression Scale [12].

### Statistical analysis

General characteristics of study participants across different levels of water intake were expressed as means ±SDs for continuous variables and percentages for categorical variables. To examine the differences across categories of water intake, we used ANOVA for continuous variables and chi-square test for categorical variables. We used binary logistic regression to estimate ORs and 95% CIs for the presence of IBS across categories of water intake in crude and multivariable-adjusted models. Age (continuous), and sex (male/female) were adjusted for in the first model. Further adjustment was done for marital status (married/single/divorced and widowed), smoking status (non-smoker/ex-smoker/current smoker), depression (yes/no), dietary supplement use (yes/no), antipsychotic medications (yes/no), and GI medications (yes/no) were controlled for in the second model. Meal regularity (often or always/never or sometimes) and dental status (having all teeth/lost 1–5 teeth/lost>5 teeth) were controlled for in the third model. P for trends was determined by considering categories of whole-day water intake as ordinal variables in the logistic regression analysis. All statistical analyses were done using the Statistical Package for Social Sciences (version 20; SPSS Inc.). P<0.05 was considered as statistically significant.

## Results

Characteristics of study participants across categories of whole-day water intake are provided in **Table 1**. Compared with those who consumed <2 glasses of water in a day, participants who were taking >8 glasses of water in a day were younger, less likely to be females, married and depressed, had more regular meal pattern, and more likely to lose 1–5 teeth. No significant differences were observed in terms of other variables across categories of water intake.

**Table 1:** General characteristics of study participants across categories of whole-day water intake^a^.

	Whole-day water intake (n. of glasses/d)				
	<2	2–5	6–8	>8	P-value^b^
Age, y	37.7±7.5	36.4±8.1	35.2±8.5	35.9±9.1	<0.001
BMI, kg/m^2^	24.7±4.7	24.5±4.0	25.07±4.7	25.01±4.9	0.17
Female, %	69.9	55	44.4	38.3	<0.001
Obesity, %	41.4	40.7	42.8	41	0.90
Married, %	81.8	82.3	79.2	71.8	<0.001
Current smokers, %	4.8	3.6	2.4	3.3	0.31
Supplement use, %	7.1	7.8	7.3	6.8	0.86
GI medications use, %	24.4	22.8	22.3	26.8	0.35
Antipsychotic medications use, %	5.1	4.9	4.0	6.8	0.33
Regular meal pattern, %					<0.001
Often or always	52.3	59.3	63.6	58.9	
Never or occasionally	47.7	40.7	36.4	41.1	
Tooth loss, %					<0.001
Have all teeth	27.7	34.9	35.5	33	
Lost 1–5 teeth	60	57.7	57.7	60.7	
Lost >5 teeth	12.2	7.3	6.8	6.3	
Depression, %	32.3	50.7	12.8	4.2	<0.001

^a^Data are mean ± standard deviation (SD) or percent.

^b^Obtained from ANOVA or chi-square test, where appropriate.

Crude and multivariable-adjusted odds ratios (ORs) and 95% confidence intervals (CIs) for IBS across categories of whole-day water intake are shown in **Table 2**. In the crude model, subjects who consumed >8 glasses of water daily had 34% lower odds of IBS (OR: 0.66; 95% CI: 0.46–0.97) than those who consumed <2 glasses; however, this association became non-significant after further adjustment for potential confounders (OR: 1.14; 95% CI: 0.74–1.78).

**Table 2:** ORs and 95% CIs for IBS across categories of whole-day water intake^a^.

	Whole-day water intake (n. of glasses/d)				
	<2	2–5	6–8	>8	P-trend
Crude	1.00	0.98 (0.83–1.15)	0.90 (0.72–1.18)	0.66 (0.46–0.97)	0.05
Model I^b^	1.00	1.01 (0.84–1.21)	0.94 (0.74–1.19)	0.84 (0.56–1.25)	0.41
Model II^c^	1.00	1.04 (0.85–1.28)	1.14 (0.87–1.49)	1.07 (0.69–1.64)	0.39
Model III^d^	1.00	1.05 (0.85–1.29)	1.17 (0.89–1.54)	1.14 (0.74–1.78)	0.26

^a^Values are OR (95% CIs).

^b^Adjusted for age and sex.

^c^Adjusted for marital status, smoking status, supplement use, depression, GI medications use and antipsychotic medications use.

^d^Additionally, adjusted for meal pattern regularity and dental status.

IBS: Irritable Bowel Syndrome.

Gender- and BMI-stratified crude and multivariable-adjusted ORs and 95% CIs for IBS across categories of whole-day water intake are shown in **Table 3**. When potential confounders were taken into account, no significant association was observed between water intake and odds of IBS in either gender [for men: OR: 1.15; 95% CI: 0.59–2.24 and for women: OR: 1.15; 95% CI: 0.62–2.12]. By BMI status, normal weight subjects (BMI<25 kg/m^2^) who were taking >8 glasses of water daily had lower odds of IBS compared with those who consumed <2 glasses (OR: 0.52; 95% CI: 0.29–0.95). However, this association became non-significant in the fully adjusted model (OR: 1.01; 95% CI: 0.51–2.01). No significant association was observed between whole day water intake and odds of IBS among overweight and obese people (BMI≥25 kg/m^2^).

**Table 3:** Gender- and BMI-stratified ORs and 95% CIs for IBS across categories of whole-day water intake^a^.

	Whole-day water intake (n. of glasses/d)				
	<2	2–5	6–8	>8	P-trend
**Male**					
Crude	1.00	1.04 (0.76–1.42)	1.14 (0.80–1.62)	0.72 (0.42–1.25)	0.72
Model I^b^	1.00	1.00 (0.71–1.41)	1.04 (0.69–1.56)	0.94 (0.51–1.70)	0.98
Model II^c^	1.00	1.21 (0.81–1.80)	1.46 (0.91–2.32)	1.10 (0.57–2.10)	0.31
Model III^d^	1.00	1.22 (0.81–1.85)	1.51 (0.93–2.43)	1.15 (0.59–2.24)	0.24
**Female**					
Crude	1.00	1.03 (0.84–1.26)	0.84 (0.62–1.13)	0.78 (0.45–1.33)	0.24
Model I^b^	1.00	1.03 (0.83–1.27)	0.85 (0.62–1.17)	0.74 (0.42–1.32)	0.26
Model II^c^	1.00	1.00 (0.78–1.27)	1.00 (0.71–1.41)	1.11 (0.60–2.05)	0.83
Model III^d^	1.00	0.99 (0.78–1.27)	0.99 (0.70–1.41)	1.15 (0.62–2.12)	0.82
**BMI<25 (kg/m**^**2**^**)**					
Crude	1.00	0.95 (0.75–1.20)	0.90 (0.66–1.22)	0.52 (0.29–0.95)	0.09
Model I^e^	1.00	0.98 (0.76–1.25)	0.90 (0.63–1.27)	0.77 (0.41–1.43)	0.38
Model II^c^	1.00	1.05 (0.79–1.39)	1.14 (0.78–1.66)	0.98 (0.51–1.87)	0.65
Model III^d^	1.00	1.06 (0.79–1.42)	1.15 (0.78–1.70)	1.01 (0.51–2.01)	0.58
**BMI≥25 (kg/m**^**2**^**)**					
Crude	1.00	1.03 (0.80–1.33)	0.87 (0.63–1.21)	0.79 (0.47–1.31)	0.92
Model I^e^	1.00	1.07 (0.82–1.41)	0.94 (0.65–1.34)	0.89 (0.50–1.57)	0.63
Model II^c^	1.00	1.05 (0.77–1.44)	1.16 (0.78–1.73)	1.19 (0.64–2.19)	0.41
Model III^d^	1.00	1.07 (0.77–1.48)	1.19 (0.79–1.79)	1.30 (0.70–2.43)	0.29

^a^Values are OR (95% CIs).

^b^Adjusted for age.

^c^Additionally, adjusted for marital status, smoking status, supplement use, depression, GI medications use and antipsychotic medications use.

^d^Additionally, adjusted for meal pattern regularity and dental status.

^e^Adjusted for age and sex.

IBS: Irritable Bowel Syndrome; BMI: Body Mass Index.

Crude and multivariable-adjusted ORs and 95% CIs for IBS subtypes across categories of whole-day water intake are shown in **Table 4.** We observed that participants who were taking >8 glasses of water daily had lower odds of IBS-C compared with those who were taking <2 glasses (OR: 0.36; 95% CI: 0.16–0.79); however, this association became non-significant in the fully adjusted model (OR: 0.51; 95% CI: 0.20–1.30). We also observed that participants who were taking >8 glasses of water daily had greater odds of IBS-M compared with those who consumed <2 glasses daily (OR: 2.07; 95% CI: 1.01–4.22). No other association was seen between water intake and other types of IBS.

**Table 4:** Crude and multivariable-adjusted ORs and 95% CIs for IBS subgroups across categories of whole-day water intake^a^.

	Whole-day water intake (n. of glasses/d)				
	<2	2–5	6–8	>8	P-trend
**IBS-C**					
Crude	1.00	1.03 (0.80–1.34)	0.69 (0.48–1.00)	0.36 (0.16–0.79)	0.004
Model I^b^	1.00	1.17 (0.88–1.54)	0.76 (0.50–1.15)	0.48 (0.20–1.12)	0.09
Model II^c^	1.00	1.14 (0.84–1.54)	0.82 (0.52–1.28)	0.58 (0.24–1.37)	0.24
Model III^d^	1.00	1.13 (0.83–1.55)	0.86 (0.55–1.35)	0.51 (0.20–1.30)	0.25
**IBS-D**					
Crude	1.00	0.83 (0.59–1.16)	0.95 (0.62–1.44)	0.87 (0.44–1.73)	0.71
Model I^b^	1.00	0.79 (0.54–1.13)	0.91 (0.56–1.45)	1.10 (0.54–2.22)	0.94
Model II^c^	1.00	0.99 (0.65–1.51)	1.32 (0.79–2.22)	1.58 (0.75–3.32)	0.14
Model III^d^	1.00	0.98 (0.64–1.50)	1.34 (0.79–2.25)	1.69 (0.80–3.57)	0.10
**IBS-M**					
Crude	1.00	0.94 (0.66–1.34)	0.93 (0.59–1.46)	1.46 (0.79–2.69)	0.55
Model I^b^	1.00	0.85 (0.59–1.24)	0.89 (0.55–1.46)	1.39 (0.71–2.71)	0.70
Model II^c^	1.00	0.84 (0.56–1.27)	1.01 (0.59–1.72)	1.64 (0.81–3.39)	0.32
Model III^d^	1.00	0.93 (0.60–1.43)	1.12 (0.65–1.95)	2.07 (1.01–4.22)	0.11
**IBS-U**					
Crude	1.00	1.07 (0.79–1.44)	1.18 (0.81–1.71)	0.51 (0.23–1.14)	0.70
Model I^b^	1.00	1.13 (0.82–1.56)	1.25 (0.83–1.89)	0.62 (0.26–1.46)	0.88
Model II^c^	1.00	1.07 (0.75–1.52)	1.36 (0.88–2.12)	0.72 (0.30–1.73)	0.57
Model III^d^	1.00	1.02 (0.72–1.46)	1.28 (0.81–2.00)	0.72 (0.30–1.74)	0.73

^a^Values are OR (95% CIs).

^b^Adjusted for age and sex.

^c^Additionally adjusted for marital status, smoking status, supplement use, depression, GI medications use and antipsychotic medications use.

^d^Additionally, adjusted for meal pattern regularity and dental status.

IBS: Irritable Bowel Syndrome; IBS-C: IBS with constipation; IBS-D: IBS with diarrhea; IBS-M: mixed IBS; IBS-U: unsubtyped IBS.

## Discussion

In this cross-sectional study, we examined the association between daily water intake and odds of IBS. We found no significant association between whole-day water intake and odds of IBS; however, there was a significant positive association between water intake and odds of IBS-M. To our knowledge, this is the first study examining the association between whole-day water intake and odds of IBS.

IBS is associated with decreased quality of life [13], therefore, management of this syndrome should be a priority of health care system. Water has numerous critical roles in the human body and optimal functioning of the human body requires a good hydration level [14]. In this study, we found no significant association between whole-day water consumption and odds of IBS. No previous study examined the association between water intake and odds of IBS; however, a clinical trial investigated the effects of alkaline-reduced water consumption on IBS-D and showed that drinking alkaline-reduced water for 8 weeks improves the quality of life in patients with IBS-D [15]. The association of water intake and other chronic diseases has been investigated in previous studies. For instance, high total water intake was protectively associated with chronic kidney disease [9]. However, cardiovascular disease was not associated with water consumption. High water consumption was also prospectively associated with increased risk of mortality among women [10]. Water intake might be associated with improvement of IBS through affecting GI function. For instance, it has been shown that adults who consumed 2 L of water had a significant increase in evacuation frequency and a decrease in laxative consumption [16]. In addition, higher fluid and dietary fiber intake was associated with improved constipation, as well as laxative use [17, 18]. It must be noted that our study is of cross-sectional design and causal relationship between water intake and IBS cannot be inferred. Considering the paucity of studies on the association of water intake and gastrointestinal health, further studies especially with prospective design are warranted in this field.

Our study had some strengths. This is the first study that examined the association of whole-day water intake and odds of IBS. Large sample size and taking the role of potential confounders into account are some strengths of the present study. In addition, dietary habits which contribute to gastrointestinal disorders were considered as covariates in our analyses. Some limitations should also be considered. To examine water consumption, we used two simple questions. Although we used a pre-tested questionnaire, misclassification of study participants in terms of exposure cannot be ignored. Although the validity of Rome III questionnaire has been shown in Iranian adults, the possibility of misclassification in terms of having IBS cannot be avoided. We collected dietary intakes of study participants in this study. Previous studies have shown that dietary intake of polyphenols might be associated with odds of IBS [19]. However, due to lack of information regarding flavonoids in the Iranian food composition database, we could not calculate the amount of polyphenols consumed by study participants. There is a recent meta-analysis which reported potentially beneficial effects of curcumin on IBS symptoms [20]. In terms of curcumin, we did not collect information on curcumin separately. However, we asked participants to report their consumption of spices within their meals (chili pepper, curry, ginger, cinnamon, and turmeric) throughout the week. Our previous study revealed that consumption of spicy foods was directly associated with IBS, particularly in women [21]. Therefore, we did not have information on curcumin separately to be able to control for that. As the study subjects were Iranian adults who were apparently healthy, the results of our study may not be generalizable to other individuals. Therefore, interpretation of our findings must be done cautiously. Further studies especially with prospective design are warranted to shed light on this issue. We collected information on other beverages (like tea, coffee, cola, etc) in SEPAHAN. However, as we aimed to examine the association of plain water with IBS, we did not include their information here. In addition, the association of other beverages with IBS might be different from that of plain water, because beverages, other than water, contain some other components, like caffeine and sugar that may affect IBS. It is suggested that future studies examine consumption of other beverages in relation to IBS.

In conclusion, we found that whole-day water intake was not associated with odds of IBS. A significant positive association between >8 glasses of water consumption daily and odds of IBS-M was observed.
